# Infectious syphilis in women and heterosexual men in major Australian cities: sentinel surveillance data, 2011–2019

**DOI:** 10.5694/mja2.51864

**Published:** 2023-02-28

**Authors:** Allison Carter, Hamish McManus, James S Ward, Tobias Vickers, Jason Asselin, Greta Baillie, Eric PF Chow, Marcus Y Chen, Christopher K Fairley, Christopher Bourne, Anna McNulty, Phillip Read, Kevin Heath, Nathan Ryder, Jenny McCloskey, Christopher Carmody, Heather McCormack, Kate Alexander, Dawn Casey, Mark Stoove, Margaret E Hellard, Basil Donovan, Rebecca J Guy

**Affiliations:** ^1^ The Kirby Institute Sydney NSW; ^2^ Australian Human Rights Institute Sydney NSW; ^3^ The University of Queensland Brisbane QLD; ^4^ Centre for Population Health Burnet Institute Melbourne VIC; ^5^ Melbourne Sexual Health Centre Alfred Health Melbourne VIC; ^6^ Central Clinical School Monash University Melbourne VIC; ^7^ New South Wales Ministry of Health Sydney NSW; ^8^ Sydney Sexual Health Centre Sydney Hospital Sydney NSW; ^9^ University of New South Wales Sydney NSW; ^10^ South Eastern Sydney Local Health District Sydney NSW; ^11^ Hunter New England Sexual Health Pacific Clinic Newcastle NSW; ^12^ St John of God Mount Lawley Medical Centre Perth WA; ^13^ South Western Sydney Local Health District Sydney NSW; ^14^ National Aboriginal Community Controlled Health Organisation Canberra ACT; ^15^ The Burnet Institute Melbourne VIC

**Keywords:** Gender identity, Sexually transmitted diseases

## Abstract

**Objectives:**

To examine changes in the positive infectious syphilis test rate among women and heterosexual men in major Australian cities, and rate differences by social, biomedical, and behavioural determinants of health.

**Design, setting:**

Analysis of data extracted from de‐identified patient records from 34 sexual health clinics participating in the Australian Collaboration for Coordinated Enhanced Sentinel Surveillance of Sexually Transmissible Infections and Blood Borne Viruses (ACCESS).

**Participants:**

First tests during calendar year for women and heterosexual men aged 15 years or more in major cities who attended ACCESS sexual health clinics during 2011–2019.

**Main outcome measures:**

Positive infectious syphilis test rate; change in annual positive test rate.

**Results:**

180 of 52 221 tested women (0.34%) and 239 of 36 341 heterosexual men (0.66%) were diagnosed with infectious syphilis. The positive test rate for women was 1.8 (95% confidence interval [CI], 0.9–3.2) per 1000 tests in 2011, 3.0 (95% CI, 2.0–4.2) per 1000 tests in 2019 (change per year: rate ratio [RR], 1.12; 95% CI, 1.01–1.25); for heterosexual men it was 6.1 (95% CI, 3.8–9.2) per 1000 tests in 2011 and 7.6 (95% CI, 5.6–10) per 1000 tests in 2019 (RR, 1.10; 95% CI, 1.03–1.17). In multivariable analyses, the positive test rate was higher for women (adjusted RR [aRR], 1.85; 95% CI, 1.34–2.55) and heterosexual men (aRR, 2.39; 95% CI, 1.53–3.74) in areas of greatest socio‐economic disadvantage than for those in areas of least socio‐economic disadvantage. It was also higher for Indigenous women (aRR, 2.39; 95% CI, 1.22–4.70) and for women who reported recent injection drug use (aRR, 4.87; 95% CI, 2.18–10.9) than for other women; it was lower for bisexual than heterosexual women (aRR, 0.48; 95% CI, 0.29–0.81) and for women who reported recent sex work (aRR, 0.35; 95% CI, 0.29–0.44). The positive test rate was higher for heterosexual men aged 40–49 years (aRR, 2.11; 95% CI, 1.42–3.12) or more than 50 years (aRR, 2.36; 95% CI, 1.53–3.65) than for those aged 15–29 years.

**Conclusion:**

The positive test rate among both urban women and heterosexual men tested was higher in 2019 than in 2011. People who attend reproductive health or alcohol and drug services should be routinely screened for syphilis.

**The known:** The prevalence of syphilis in Australia is higher among men who have sex with men and non‐urban Aboriginal and Torres Strait Islander people.

**The new:** Among people tested at sexually transmitted infection clinics in major Australian cities, the positive syphilis test rate was higher for both women and heterosexual men in 2019 than in 2011. The rate was higher among people living in areas of greater socio‐economic disadvantage, and also for Indigenous women and women who inject drugs.

**The implications:** Screening people for syphilis in reproductive health and alcohol and drug services is important, as is attention to the social determinants of syphilis risk.

The annual number of notifications of infectious syphilis has almost quadrupled during the past decade in Australia, from 1332 notifications in 2011 to 5248 in 2020.^1^ Similar rises have been reported in Canada,^2^ the United Kingdom,^3^ the United States,^4^ Europe,^5^ and Asia.^6^, ^7^ Syphilis is infectious during the first two years after infection, particularly during its primary and secondary stages; the sexual contact transmission risk is as high as 50%.^8^ Untreated infectious syphilis increases the risk of human immunodeficiency virus (HIV) infection^9^ and can have serious effects in pregnant women, including stillbirth and miscarriage.^10^ As no vaccine is available, syphilis control largely depends upon consistent condom use and early diagnosis and treatment.^11^

In Australia, the infectious syphilis notification rate has generally been highest among urban men who have sex with men^1^ and Aboriginal and Torres Strait Islander people in regional and remote areas.^12^ More recently, the number of syphilis notifications for women has risen, from 195 in 2014 to 945 in 2020.^1^ This rise suggests that heterosexual transmission is increasing, and also raises concerns about the risk of transmission during pregnancy; 32 cases of congenital syphilis were notified during 2020 and 2021.^13^

Factors that may contribute to more notifications of syphilis in heterosexual people include changes in testing patterns, sexual practices, and patterns of connection between communities in which syphilis is more prevalent.^14^ Infection may also be associated with social conditions^15^ in which inequities related to social identity (gender, ethnic background, socio‐economic status) and institutional and societal structures (poverty, racism, restricted health care access) interact, placing certain subgroups of people at greater risk of synergistic epidemics (syndemics).^16^ In Canada, for example, large proportions of women with infectious syphilis reported concurrent social and health problems, such as housing instability, income assistance, and mental illness.^15^

To inform strategies for preventing syphilis in heterosexuals in Australia, we examined in changes in the positive infectious syphilis test rate among women and heterosexual men in major Australian cities. We were particularly interested in examining differences between women and heterosexual men, as well as differences within gender groups according to social, biomedical, and behavioural determinants of health.

## Method

We analysed data extracted by the Australian Collaboration for Coordinated Enhanced Sentinel Surveillance of Sexually Transmissible Infections and Blood Borne Viruses (ACCESS; accessproject.org.au).^17^ We included data for the period 1 January 2011 – 31 December 2019 retrospectively extracted from de‐identified patient records from 34 sexual health clinics (some services include several clinics; Queensland: Gold Coast Sexual Health; New South Wales: Coffs Harbour Sexual Health Clinic, Far West NSW Sexual Health [Broken Hill, Dareton], Hunter New England Sexual Health Service, Lismore Sexual Health, Liverpool Sexual Health Clinic, Nepean and Blue Mountains Sexual Health Clinic [Katoomba], Royal Prince Alfred Hospital Community Health Sexual Health Service [Sydney], Sydney Sexual Health Centre (Sydney Hospital), Western NSW Sexual Health [Bourke, Dubbo, Orange, Lightning Ridge], Western Sydney Sexual Health Clinic [Parramatta]; Victoria: Melbourne Sexual Health Centre; Western Australia: South Terrace Clinic [Fremantle]). Data from fourteen further clinics were not included because they were incomplete. We did not assess testing during the coronavirus disease 2019 (COVID‐19) pandemic period because syphilis testing of asymptomatic people declined during this period. Patient details in records from different clinics were probabilistically linked using GRHANITE software.^18^

### Study population

The study population comprised women and heterosexual men aged 15 years or more who lived in major Australian cities and had attended a participating sexual health clinic during the study period. Gender information was collected from participants at all clinics during patient registration. We excluded people who reported being intersex (99 people) or transgender (555 people), as well as those whose sex was unknown (twelve people), because their numbers were too small for meaningful analysis.

We defined heterosexual men as men who reported they had had sexual contact only with women during the preceding twelve months, and bisexual women as women who reported sex with both men and women during the preceding twelve months.

We examined the following health determinants:

- socio‐demographic factors: age, gender, Indigenous status, country of birth (Australia, overseas), and culturally and linguistically diverse background (people born in any country other than Australia, New Zealand, Canada, Ireland, South Africa, the United States, and the United Kingdom);
- socio‐economic factors: Socio‐Economic Indexes for Areas (SEIFA) Index of Relative Socio‐Economic Advantage and Disadvantage (IRSAD) centiles;^19^
- behavioural factors: injecting drug use in the past twelve months; for women only: commercial sex work in the past twelve months and sexual orientation (based on gender of non‐paying sexual partners during the past twelve months); and
- biomedical factors: contact with a person with a sexually transmitted infection.

### Outcome

The primary outcome was the infectious syphilis positive test rate; that is, the proportion of test results (restricted to the first test in each calendar year for an individual) that led to the diagnosis of primary, secondary, or early latent syphilis (less than two years since infection). We selected this outcome rather than syphilis incidence because repeated testing for sexually transmitted infections is uncommon among heterosexual people. We included only the first test in a calendar year for each person because people at particular risk may present for testing and be diagnosed more than once within a calendar year. The positive result rate for first tests reflects disease prevalence in the tested population, and is less subject to detection bias caused by differences in rates derived from data that include repeated testing of people from populations at higher risk.

### Statistical analysis

We estimated the positive test rate by year (per 1000 persons tested, with 95% confidence interval [CI]) for women and heterosexual men separately, and estimated the annual change in rate (as rate ratios [RRs] with 95% CIs) using Poisson regression. We used robust variance estimation, adjusted for intra‐clinic correlation of repeated measures using random effects. Negative binomial models were used if data overdispersion or non‐convergence was apparent.

We assessed differences in positive test rate by characteristic in bivariate trend models adjusted for time (reported as RRs with 95% CIs); interactions of risk factors with trend were also investigated. We then undertook analyses in multivariable Poisson regression models (adjusted for all other variables and for time and for the interaction between “time” and “culturally and linguistically diverse”); results are reported as adjusted RRs (aRRs) with 95% CIs.

For multivariable analyses, multicollinearity was assessed by inspecting the correlation matrix for all highly correlated variables (*r* greater than 0.7) and examining variance inflation factors and the condition index (variance inflation factors greater than 4 or condition index greater than 30 deemed potentially problematic), as well as variance decomposition (more than one covariate with decomposition proportion greater than 0.80 deemed potentially problematic^20^). No collinearity was detected.

All analyses were undertaken in Stata 15.1 for Windows.

### Ethics approval

The human research ethics committees of the Alfred Hospital (248/17), the Northern Territory Department of Health and Menzies School of Health (08/47), the University of Tasmania (H0016971), the Aboriginal Health and Medical Research Council (NSW; 1099/15), and St. Vincent's Hospital (08/051) approved the study, as did the Central Australian Human Research Ethics Committee (19‐3355), ACON (2015/14), the Victorian AIDS Council and Thorne Harbour Health (VAC REP 15/003), and the Western Australian Aboriginal Health Ethics Committee (885). Individual patient consent was not required for our analysis of de‐identified data collected for public health surveillance.

## Results

Of 144 468 people (85 772 women, 58 696 heterosexual men) from major cities who attended ACCESS sexual health clinics during 2011–2019, 88 562 (61%) were tested for syphilis (61%). The median age of the 52 221 women was 28 years (interquartile range [IQR], 24–34 years), that of the 36 341 men was 31 years (IQR, 25–38 years); 908 people were Aboriginal or Torres Strait Islander people (1.0%). The proportion of women with culturally or linguistically diverse backgrounds was larger than for men (44.4% *v* 29.9%), as was the proportion who had arrived in Australia less than two years ago (33.5% *v* 17.7%). Injecting drug use during the past twelve months was reported by 1100 women (2.1%) and 1085 men (3.0%). A total of 12 870 women reported paid sex work during the preceding twelve months (24.6%); 32 762 women described themselves as heterosexual (62.7%), 649 as homosexual (1.2%), and 3999 as bisexual (7.7%). Contacts with people with sexually transmitted infections were reported by 2820 women (5.4%) and 2561 heterosexual men (7.1%) (Box 1).

Box 1 **Characteristics of women and heterosexual men in major Australian cities at their first infectious syphilis tests in a calendar year at participating ACCESS sexual health clinics, 2011–2019**

**Table:** 

Characteristic	Women	Heterosexual men
Number of people	52 221	36 341
**Socio‐demographic and economic factors**		
Age group (years)		
15–29	30 107 (57.6%)	16 084 (44.3%)
30–39	15 216 (29.1%)	12 088 (33.3%)
40–49	5006 (9.6%)	4487 (12.4%)
50 or older	1892 (3.6%)	3682 (10.1%)
Indigenous status		
Aboriginal or Torres Strait Islander	533 (1.0%)	375 (1.0%)
Non‐Indigenous	51 688 (99.0%)	35 966 (99.0%)
Culturally or linguistically diverse background		
No	29 029 (55.6%)	25 474 (70.1%)
Yes	23 192 (44.4%)	10 867 (29.9%)
Arrival in Australia		
Born in Australia	19 981 (38.3%)	19 986 (55.0%)
Less than two years	17 471 (33.5%)	6431 (17.7%)
Two to less than five years	7617 (14.6%)	3389 (9.3%)
Five years or more	7152 (13.7%)	6535 (18.0%)
Socio‐economic status (IRSAD centile)		
81–100 [least disadvantage]	22 878 (43.8%)	14 718 (40.5%)
41–80	21 540 (41.2%)	14 353 (39.5%)
0–40 [greatest disadvantage]	7803 (14.9%)	7270 (20.0%)
**Behavioural factors**		
Injection drug use in the past twelve months		
No	51 121 (97. 9%)	35 256 (97.0%)
Yes	1100 (2.1%)	1085 (3.0%)
Sexual orientation		
Heterosexual	32 762 (62.7%)	—
Homosexual	649 (1.2%)	—
Bisexual	3999 (7.7%)	—
Missing data	14 811 (28.4%)	—
Sex work in the past twelve months		
No	39 351 (75.4%)	—
Yes	12 870 (24.6%)	—
**Biomedical factor**		
Contact with person with a sexually transmissible infection		
No	49 401 (94.6%)	33 780 (93.0%)
Yes	2820 (5.4%)	2561 (7.1%)

ACCESS = Australian Collaboration for Coordinated Enhanced Sentinel Surveillance of Sexually Transmissible Infections and Blood Borne Viruses; IRSAD = Index of Relative Socio‐Economic Advantage and Disadvantage.^19^

### Change in positive test rates, 2011–2019

During 2011–2019, 180 women (0.34%) and 239 heterosexual men (0.66%) were diagnosed with infectious syphilis. For women, the positive test rate was 1.8 (95% CI, 0.9–3.2) per 1000 tested in 2011 and 3.0 (95% CI, 2.0–4.2) per 1000 tested in 2019 (change per year: RR, 1.12; 95% CI, 1.01–1.25); for heterosexual men it was 6.1 (95% CI, 3.8–9.2) per 1000 tested in 2011, and 7.6 (95% CI, 5.6–10) per 1000 tested in 2019 (RR, 1.10; 95% CI, 1.03–1.17) (Box 2, Box 3).

Box 2 Diagnoses of syphilis at first tests in calendar year for women and heterosexual men in major Australian cities during consultations in participating ACCESS sexual health clinics, 2011–2019*

**Table:** 

	Women		Heterosexual men	
Year	Positive tests/all tests	Number per 1000 tests (95% CI)	Positive tests/all tests	Number per 1000 tests (95% CI)
2011	11/6054 (0.18%)	1.8 (0.9–3.2)	22/3631 (0.61%)	6.1 (3.8–9.2)
2012	8/6364 (0.1%)	1.3 (0.5–2.5)	9/4233 (0.2%)	2.1 (1.0–4.0)
2013	16/6891 (0.23%)	2.3 (1.3–3.8)	22/4477 (0.49%)	4.9 (3.1–7.4)
2014	12/7540 (0.16%)	1.6 (0.8–2.8)	19/4868 (0.39%)	3.9 (2.4–6.1)
2015	13/7709 (0.17%)	1.7 (0.9–2.9)	26/4563 (0.57%)	5.7 (3.7–8.3)
2016	22/7706 (0.29%)	2.9 (1.8–4.3)	25/4921 (0.51%)	5.1 (3.3–7.5)
2017	32/8274 (0.39%)	3.9 (2.6–5.5)	29/5091 (0.57%)	5.7 (3.8–8.2)
2018	34/9892 (0.34%)	3.4 (2.4–4.8)	37/6425 (0.58%)	5.8 (4.1–7.9)
2019	32/10 756 (0.30%)	3.0 (2.0–4.2)	50/6612 (0.76%)	7.6 (5.6–10)

ACCESS = Australian Collaboration for Coordinated Enhanced Sentinel Surveillance of Sexually Transmissible Infections and Blood Borne Viruses; CI = confidence interval.

* Positive test results/total number of first time clinic attenders tested.

Box 3 Positive syphilis test rate for women and heterosexual men in major Australian cities for first tests in calendar year at participating ACCESS sexual health clinics, 2011–2019*

**Figure:**
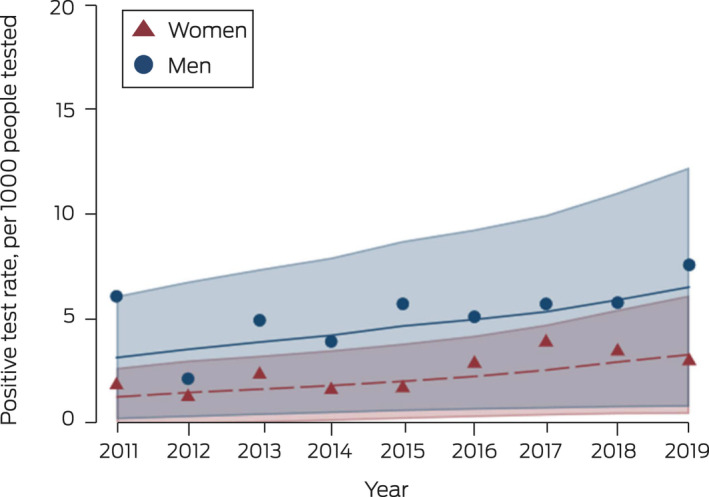
ACCESS = Australian Collaboration for Coordinated Enhanced Sentinel Surveillance of Sexually Transmissible Infections and Blood Borne Viruses. * Scatter plot: calculated annual positive test rate. Line plot: fitted annual rate of change, adjusted for patient‐level random effects and time, with 95% confidence region.

For women, the largest changes in the positive test rate were for those who reported injecting drug use during the past twelve months (from 7.2 [95% CI, 6.9–21] to 28 [95% CI, 3.4–52] per 1000 women tested), Australian‐born women (from 2.3 [95% CI, 0.5–4.2] to 5.9 [95% CI, 3.5–8.3] per 1000 women tested), and Aboriginal and Torres Strait Islander women (from 19 [95% CI, 18–56] to 47 [95% CI, 1–93] per 1000 women tested). For heterosexual men, the largest changes were for those who reported injecting drug use during the past twelve months (from 6.0 [95% CI, 3.6–6.6] to 19 [95% CI, 2.5–41] per 1000 men tested) and Australian‐born men (from 5.4 [95% CI, 2.3–8.5] to 11 [95% CI, 7.7–15] per 1000 men tested) (Supporting Information, figures 1 to 7).

### Positive test rates by characteristic

In multivariable analyses, the positive test rate was higher for women (aRR, 1.85; 95% CI, 1.34–2.55) and heterosexual men (aRR, 2.39; 95% CI, 1.53–3.74) from areas of greatest socio‐economic disadvantage than for those from areas of least disadvantage. It was higher for Indigenous than non‐Indigenous women (aRR, 2.39; 95% CI, 1.22–4.70) and for women who reported injecting drug use during the past twelve months (aRR, 4.87; 95% CI, 2.18–10.9); it was lower for bisexual than heterosexual women (aRR, 0.48; 95% CI, 0.29–0.81) and for women who reported sex work during the past twelve months (aRR, 0.35; 95% CI, 0.29–0.44). The positive test rate was higher for heterosexual men aged 40–49 years (aRR, 2.11; 95% CI, 1.42–3.12) or more than 50 years (aRR, 2.36; 95% CI, 1.53–3.65) than for men aged 15–29 years. At the start of the analysis period (2011), the positive test rate was higher for women (aRR, 3.72; 95% CI, 1.41–9.81) but not men from culturally and linguistically diverse backgrounds (aRR, 2.29; 95% CI, 0.81–6.42). The rate of infectious syphilis among people from culturally and linguistically diverse backgrounds did not change during 2011–2019, but increased by 21% among people without culturally and linguistically diverse backgrounds (Box 4).

Box 4 Positive infectious syphilis test rate at first tests in calendar year for women and heterosexual men in major Australian cities at participating ACCESS sexual health clinics, 2011–2019, by characteristic: univariable and multivariable Poisson regression models*

**Table:** 

	Women		Heterosexual men	
Characteristic	Rate ratio^†^ (95% CI)	Adjusted rate ratio (95% CI)^‡^	Rate ratio^†^ (95% CI)	Adjusted rate ratio (95% CI)^§^
Time (per year)	1.12 (1.00–1.25)	1.21 (1.06–1.37)	1.10 (1.04–1.17)	1.17 (1.09–1.25)
**Socio‐demographic and economic factors**				
Age group (years)				
15–29	1	1	1	1
30–39	0.98 (0.56–1.72)	1.00 (0.53–1.90)	1.41 (0.80–2.49)	1.32 (0.82–2.11)
40–49	1.40 (0.95–2.06)	1.27 (0.93–1.75)	2.58 (1.42–4.70)	2.11 (1.42–3.12)
50 or more	1.37 (0.63–2.96)	1.21 (0.63–3.39)	2.85 (1.66–4.90)	2.36 (1.53–3.65)
Indigenous Australian	4.19 (2.36–7.43)	2.39 (1.22–4.70)	0.69 (0.20–2.42)	0.49 (0.15–1.61)
Socio‐economic status (IRSAD centile)				
81–100 [least disadvantage]	1	1	1	1
41–80	0.87 (0.67–1.14)	0.89 (0.65–1.21)	1.39 (0.99–1.94)	1.37 (1.02–1.85)
0–40 [greatest disadvantage]	2.01 (1.37–2.93)	1.85 (1.34–2.55)	2.57 (1.44–4.57)	2.39 (1.53–3.74)
Culturally or linguistically diverse background	0.61 (0.27–1.37)	3.72 (1.41–9.81)	0.75 (0.47–1.21)	2.28 (0.81–6.41)
Arrival in Australia				
Born in Australia	1	1	1	1
Less than two years	0.28 (0.10–0.74)	0.24 (0.09–0.64)	0.33 (0.24–0.46)	0.39 (0.32–0.47)
Two to less than five years	0.36 (0.16–0.84)	0.33 (0.15–0.73)	0.22 (0.08–0.62)	0.24 (0.11–0.54)
Five years or more	0.48 (0.21–1.07)	0.41 (0.20–0.84)	0.75 (0.66–0.85)	0.62 (0.50–0.78)
**Behavioural factors**				
Injecting drug use in the past twelve months	5.75 (2.94–11.3)	4.87 (2.18–10.9)	2.33 (1.01–5.37)	1.96 (1.00–3.87)
Sexual orientation				
Heterosexual	1	1	—	—
Homosexual	1.03 (0.44–2.39)	0.83 (0.31–2.23)	—	—
Bisexual	0.53 (0.33–0.84)	0.48 (0.29–0.81)	—	—
Missing data	0.80 (0.50–1.29)	0.96 (0.58–1.59)	—	—
Sex work in the past twelve months	0.36 (0.28–0.46)	0.35 (0.29–0.44)	—	—
**Biomedical factors**				
Contact with person with a sexually transmissible infection	4.50 (3.45–5.88)	4.09 (3.16–5.29)	1.33 (0.82–2.16)	1.52 (0.94–2.48)

ACCESS = Australian Collaboration for Coordinated Enhanced Sentinel Surveillance of Sexually Transmissible Infections and Blood Borne Viruses; CI = confidence interval; IRSAD = Index of Relative Socio‐Economic Advantage and Disadvantage.^19^

* Bivariate models include an independent time covariate, except for “culturally or linguistically diverse background”, which included a time interaction covariate.

† Models were adjusted for time (per year). All covariates were tested for interaction with time; this was statistically significant only for the “culturally and linguistically diverse” variable.

‡ Adjusted for all other variables in this table; adjusted for “time” (rate ratio, 1.21; 95% CI, 1.06–1.37); and adjusted for interaction of “culturally or linguistically diverse background” and “time” (rate ratio, 0.82; 95% CI, 0.71–0.95).

§ Adjusted for all other variables in this table; adjusted for “time” (rate ratio, 1.17; 95% CI, 1.09–1.25); and adjusted for interaction of “culturally or linguistically diverse background” and “time” (rate ratio, 0.86; 95% CI, 0.74–0.99).

## Discussion

We found that infectious syphilis positive test rates (first tests in calendar year only) for women and heterosexual men tested in ACCESS sexual health clinics in major Australian cities increased during 2011–2019. The rate was higher for Aboriginal or Torres Strait Islander women, women living in lower socio‐economic status areas, and women who reported recent injecting drug use than for other tested women, and for men living in lower socio‐economic status areas or aged 40 years or more than for other tested heterosexual men. These findings suggest that the rise in syphilis notifications among women and heterosexual men in Australia is influenced by social and other health‐related conditions, indicating that a syndemic approach to treatment and prevention would be useful.^16^

The positive test rate was about twice as high for both heterosexual men and women living in areas of greatest disadvantage than for those in areas of least disadvantage. In Canada, large proportions of women diagnosed with syphilis reported negative social conditions, including housing instability and need for income assistance.^15^ Injection drug use was not frequent among the people in our study, but was associated with higher positive test rates among women. This finding is consistent with a report of the elevated risk of sexually transmitted infections for women in heterosexual relationships in which injection drugs are used.^21^

The positive test rate was higher for Indigenous than non‐Indigenous women, but the magnitude of the difference was reduced by adjustment for other factors, suggesting that other social determinants of health included in our model increase the risk of sexually transmitted infection risk for Aboriginal and Torres Strait Islander women. Sexual health differences between Indigenous and non‐Indigenous Australians are linked with adverse social conditions that can exacerbate structural obstacles to health care, such as intergenerational trauma, substance use, mental illness, poverty, incarceration, and social exclusion.^22^ We similarly found that the positive test rate was higher for women with culturally or linguistically diverse backgrounds at the start of the analysis and across the study period.

The higher positive test rates for heterosexual men over 40 years of age than for younger men could be related to differences in travel opportunities for older men and in condom use in countries where the prevalence of syphilis is high, as was also reported for gonorrhoea and HIV infection in the 1990s.^23^, ^24^

Finally, sex work was not associated with higher positive test rates in women, challenging popular perceptions of risk. This finding is, however, consistent with the report that the prevalence of sexually transmitted infections in Victoria is extremely low among female sex workers who are tested regularly.^25^ As 25% of the women in our study reported sex work during the preceding twelve months, and the positive test rate for this subgroup was lower than for other women, the increase in prevalence of infectious syphilis in the general female population is probably greater than we estimated.

### Limitations

First, generalising our findings beyond the sentinel urban settings we assessed should be cautious. Analysing results for first tests reduce the bias associated with repeat testing of people with higher sexually transmitted infection risks, but risk in our study group will be different from that of the general population. Second, as only incomplete data were available for behavioural factors such as number of sexual partners and condom use, we did not include these variables in our analyses. Third, as we did not include cases identified in general practices, because these services do not report syphilis stage, we may have underestimated case numbers. However, many people with syphilis seen in general practice would be referred to sexual health clinics for treatment or contact management, and would be included in our analysis because it is standard practice to re‐test if there is a considerable lag between general practice testing and presentation to the sexual health clinic; if re‐testing is not undertaken, a clinical diagnostic code would flag the case. We excluded syphilis testing of people with similar clinical symptoms if they were tested only for other sexually transmitted infections. Fourth, the proportion of Aboriginal and Torres Strait Islander people in our analysis was lower than their proportion in major cities, and the corresponding findings should be interpreted with caution. Finally, we did not include other factors required for a more in‐depth syndemic analysis, such as housing, mental health, and violence.

### Conclusions

We found that first‐test diagnoses of infectious syphilis in women and heterosexual men tested in major cities in Australia increased during 2011–2019, and that the positive test rate differed by social and other health‐related factors. Testing for syphilis, timely treatment, and contact management for urban heterosexuals should be increased. Health care practitioners should consider syphilis screening (at least twice) of all pregnant women (particularly those with risk factors), syphilis screening in alcohol and drug services, and integrating syphilis testing into all sexually transmitted infection screening. As social and structural factors can hinder access to conventional health care, outreach point‐of‐care rapid tests may be more appropriate for Aboriginal and Torres Strait Island communities, people in socio‐economically disadvantaged areas, and people who inject drugs.^26^ Sexually transmitted infection care should be integrated into community‐led and culturally safe services to meet the psychosocial needs of heterosexuals at risk of syphilis in Australia.

## Open access

Open access publishing facilitated by University of New South Wales, as part of the Wiley – University of New South Wales agreement via the Council of Australian University Librarians.

## Competing interests

No relevant disclosures.

## Supporting information


Supporting Information
