# Supplementary material for: Infectious syphilis in women and heterosexual men in major Australian cities: sentinel surveillance data, 2011–2019
Source: Med J Aust. 2023 Feb 28;218(5):223–8. doi: 10.5694/mja2.51864 (PMC10952992; doi:10.5694/mja2.51864)
Supplement: Supplementary file 1 — Supporting Information [file MJA2-218-223-s001.pdf]

The Medical Journal of Australia

Supporting Information

Supplementary results

This appendix was part of the submitted manuscript and has been peer reviewed.
It is posted as supplied by the authors.

Appendix to: Carter A, McManus H, Ward JS, et al. Infectious syphilis in women and heterosexual men in major Australian cities: sentinel surveillance data, 2011–2019. *Med J Aust* 2023; doi: 10.5694/mja2.51864.

Figure 1. Annual positivity (rate per 1000 person tested) for infectious syphilis by Aboriginal and Torres Strait Islander Status among women and heterosexual men in major cities of Australia attending sexual health clinics: 2011-2019 (n=88,562)

Women | Heterosexual Men

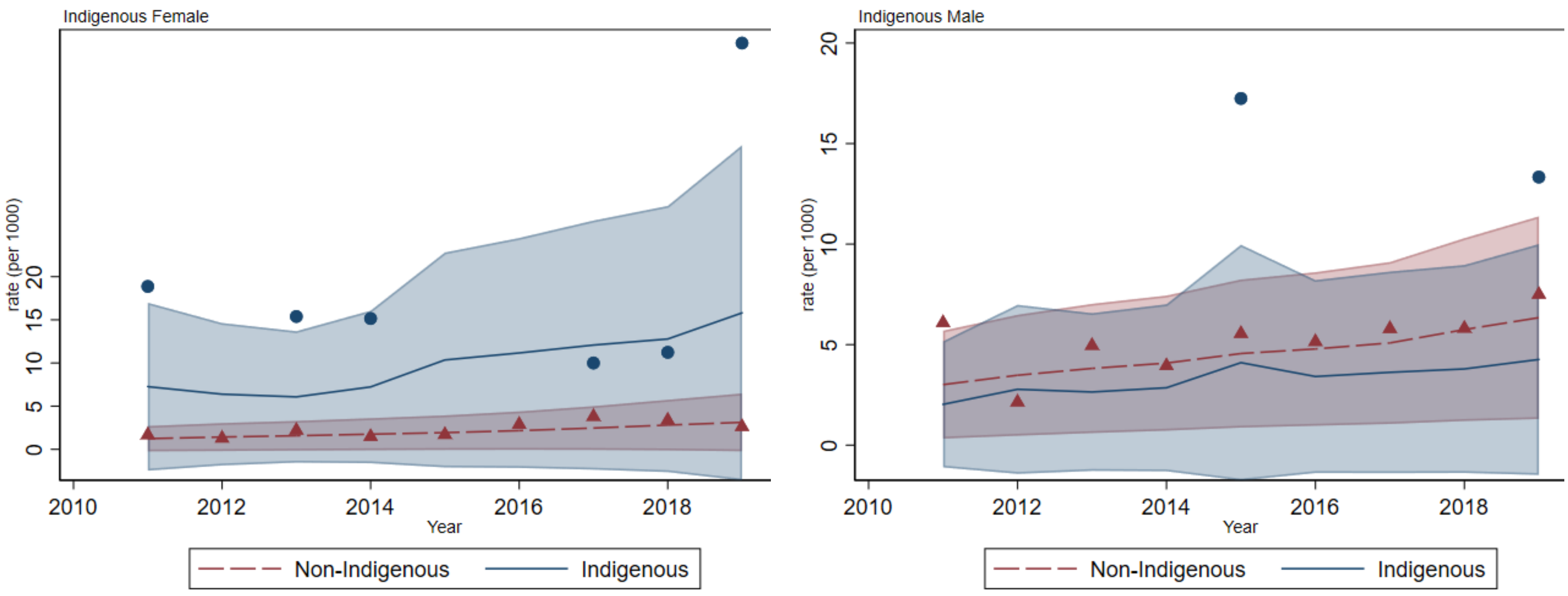

	2011	2012	2013	2014	2015	2016	2017	2018
Indigenous rates	18.9	(-)	15.4	15.2	(-)	(-)	10.0	11.2
Non-indigenous rates	1.67	1.27	2.20	1.47	1.70	2.88	3.79	3.37

	2011	2012	2013	2014	2015	2016	2017	2018	2019
Indigenous rates	(-)	(-)	(-)	(-)	17.24	(-)	(-)	(-)	13.3
Non-indigenous rates	6.09	2.14	4.95	3.95	5.55	5.15	5.79	5.81	7.50

Note: Scatter plot represents observed rates. Line plot shows fitted mean annual rate of change, adjusted for patient-level random effects and annual fluctuation.

Figure 2. Annual positivity (rate per 1000 person tested) for infectious syphilis by culturally and linguistically diverse (CALD) backgrounds among women and heterosexual men in major cities of Australia attending sexual health clinics: 2011-2019 (n=88,562)

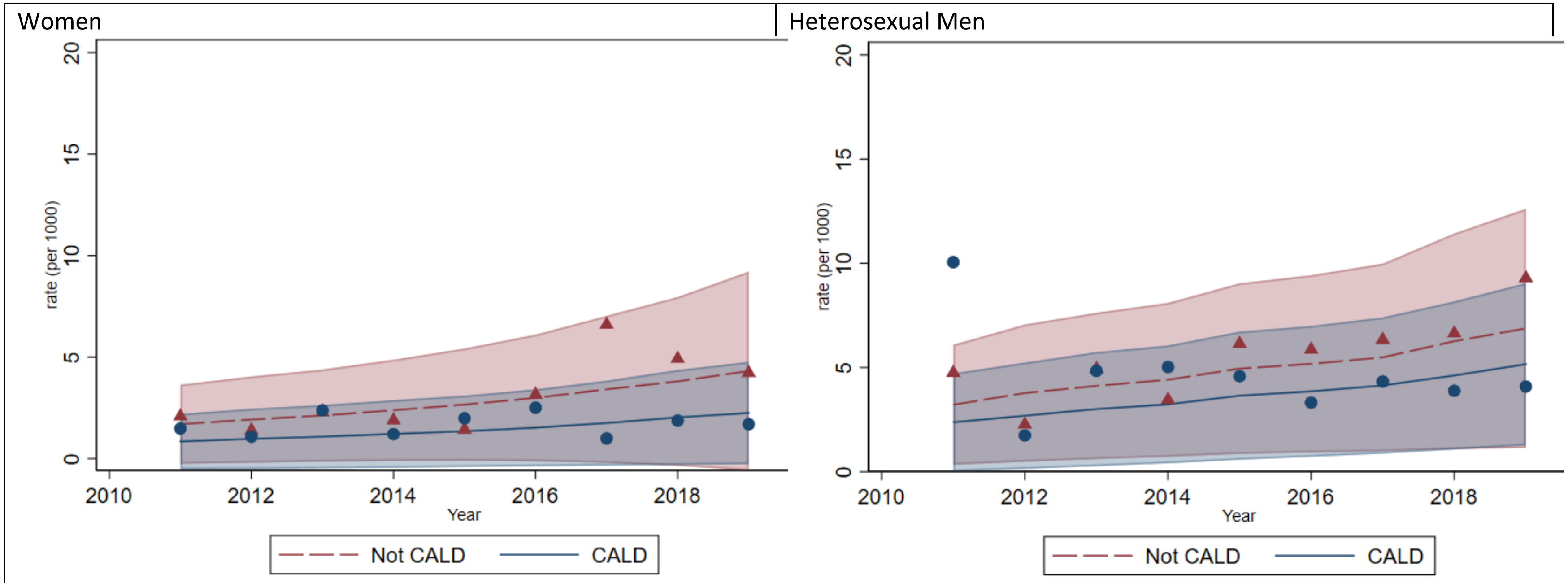

	2011	2012	2013	2014	2015	2016	2017	2018	2019
CALD rates	1.48	1.08	2.38	1.21	1.98	2.51	0.99	1.87	1.70
Non-CALD rates	2.09	1.39	2.28	1.89	1.44	3.16	6.60	4.92	4.22

	2011	2012	2013	2014	2015	2016	2017	2018
CALD rates	10.1	1.74	4.84	5.03	4.58	3.32	4.33	3.89
Non-CALD rates	4.75	2.27	4.94	3.45	6.15	5.86	6.33	6.64

Note: CALD = Culturally and Linguistically Diverse. Scatter plot represents observed rates. Line plot shows fitted mean annual rate of change, adjusted for patient-level random effects and annual fluctuation.

Figure 3. Annual positivity (rate per 1000 person tested) for infectious syphilis by year since arrival among women and heterosexual men in major cities of Australia attending sexual health clinics: 2011-2019 (n=88,562)

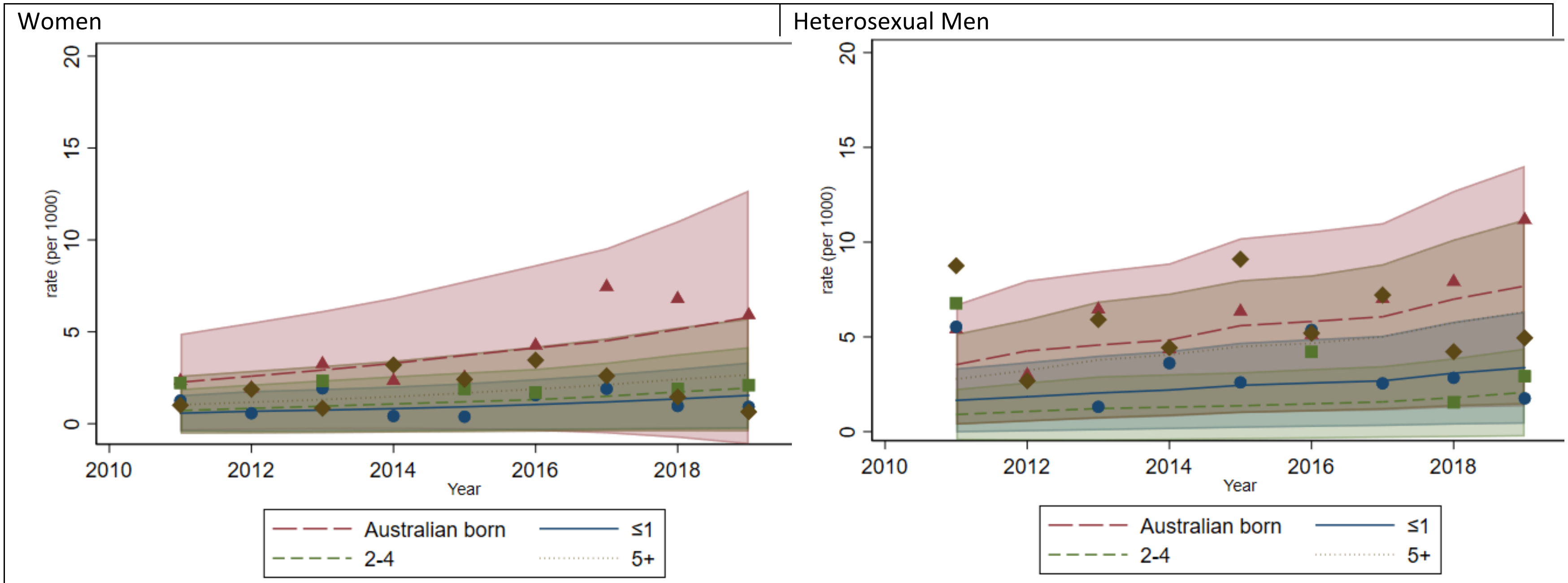

Women

	2011	2012	2013	2014	2015	2016	2017	2018	2019
Australian	2.33	1.93	3.25	2.34	2.51	4.26	7.45	6.80	5.92
<2	1.26	0.57	1.92	0.42	0.38	1.56	1.90	0.97	0.92
2-<5	2.22	(-)	2.33	(-)	1.89	1.70	(-)	1.90	2.10
≥5	1.01	1.87	0.84	3.20	2.42	3.46	2.60	1.45	0.65

Heterosexual Men

	2011	2012	2013	2014	2015	2016	2017	2018	2019
Australian	5.40	2.99	6.45	4.36	6.35	5.11	7.00	7.91	11.2
<2	5.53	(-)	1.31	3.61	2.60	5.37	2.55	2.84	1.76
2-<5	6.78	(-)	(-)	(-)	(-)	4.21	(-)	1.55	2.93
≥5	8.76	2.68	5.92	4.43	9.10	5.20	7.21	4.22	4.95

Note: Scatter plot represents observed rates. Line plot shows fitted mean annual rate of change, adjusted for patient-level random effects and annual fluctuation.

Figure 4. Annual positivity (rate per 1000 person tested) for infectious syphilis by Socio-economic Indexes for Area (SEIFA) Decile (Index of Relative Socio-Economic Advantage and Disadvantage) among women and heterosexual men in major cities of Australia attending sexual health clinics: 2011-2019 (n=88,562)

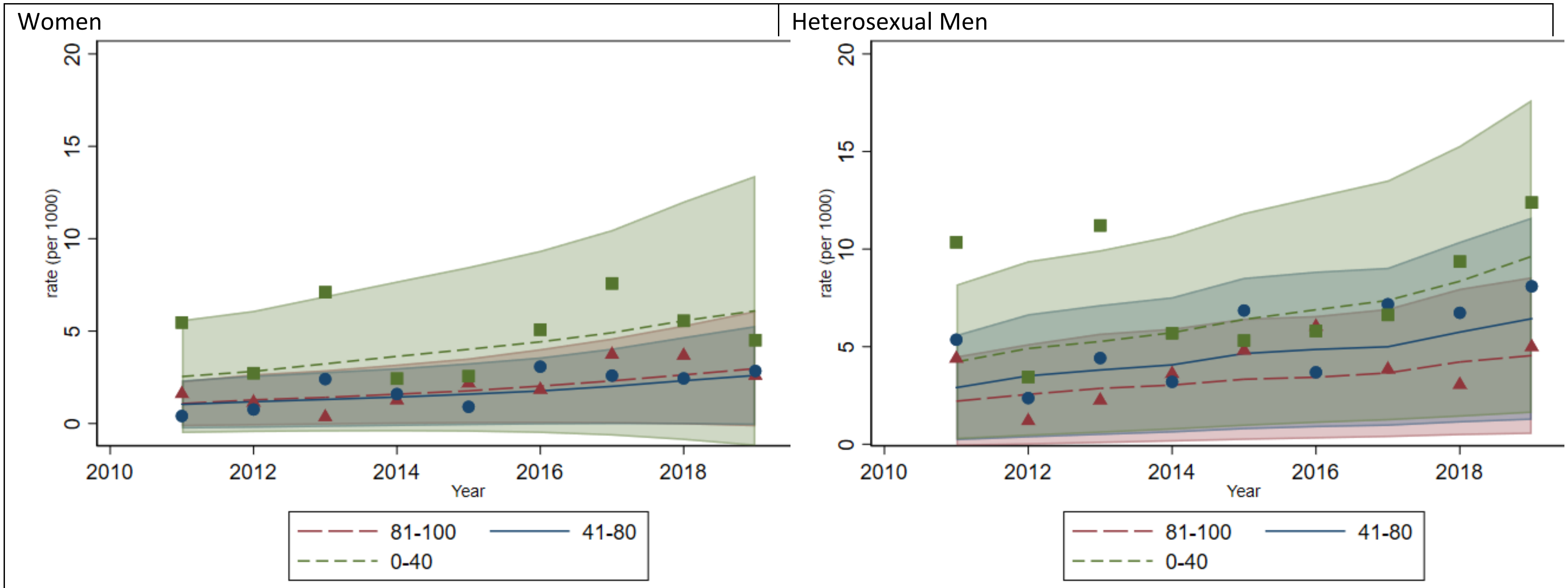

Women

	2011	2012	2013	2014	2015	2016	2017	2018	2019
81-100	1.61	1.14	0.35	1.26	2.18	1.83	3.73	3.68	2.59
41-80	0.41	0.76	2.40	1.60	0.90	3.08	2.59	2.44	2.84
0-40	5.46	2.72	7.10	2.43	2.56	5.08	7.58	5.56	4.51

Heterosexual Men

	2011	2012	2013	2014	2015	2016	2017	2018	2019
81-100	4.40	1.20	2.25	3.61	4.81	6.03	3.84	3.05	4.99
41-80	5.36	2.37	4.42	3.20	6.85	3.69	7.18	6.74	8.09
0-40	10.4	3.45	11.2	5.69	5.31	5.80	6.63	9.36	12.4

Note: Scatter plot represents observed rates. Line plot shows fitted mean annual rate of change, adjusted for patient-level random effects and annual fluctuation.

Figure 5. Annual positivity (rate per 1000 person tested) for infectious syphilis by Injecting drug use (IDU) in past 12 months among women and heterosexual men in major cities of Australia attending sexual health clinics: 2011-2019 (n=88,562)

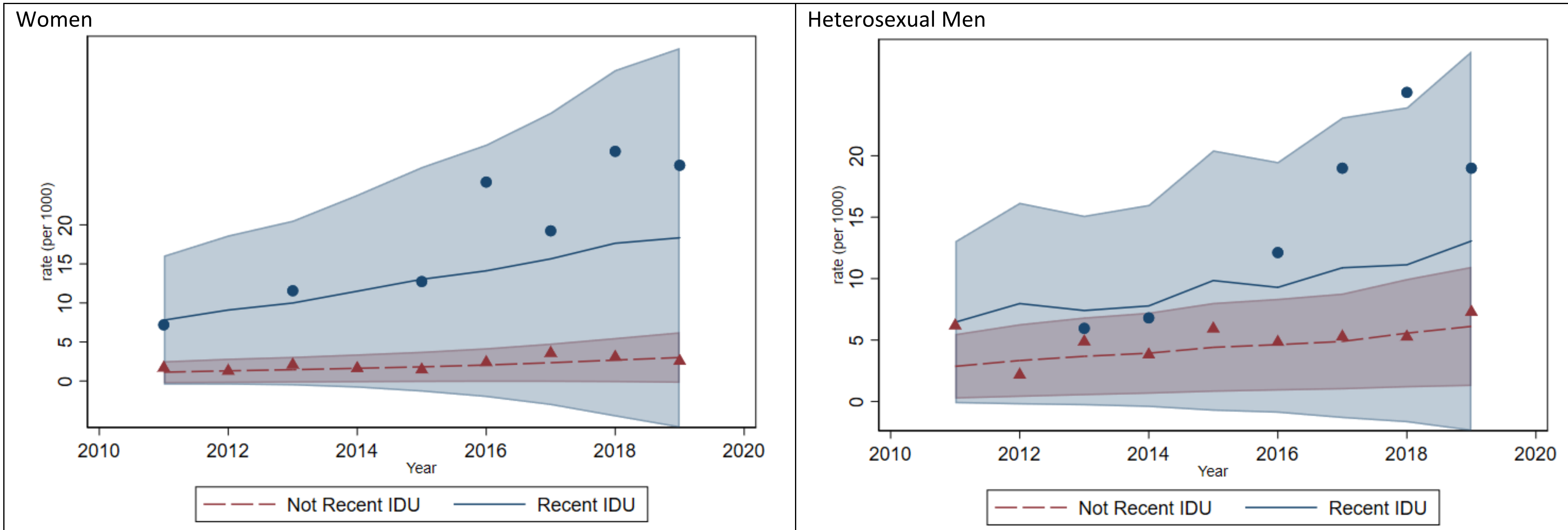

Women

	2011	2012	2013	2014	2015	2016	2017	2018	2019
Recent IDU rates	7.19	(-)	11.56	(-)	12.7	25.5	19.2	29.4	27.6
Not Recent IDU rates	1.69	1.29	2.08	1.63	1.46	2.38	3.57	3.08	2.55

Heterosexual Men

	2011	2012	2013	2014	2015	2016	2017	2018	2019
Recent IDU rates	(-)	(-)	5.95	6.80	(-)	12.1	19.0	25.2	19.0
Not Recent IDU rates	6.18	2.17	4.87	3.81	5.93	4.84	5.27	5.27	7.28

Note: IDU = Injecting Drug Use. Scatter plot represents observed rates. Line plot shows fitted mean annual rate of change, adjusted for patient-level random effects and annual fluctuation.

Figure 6. Annual positivity (rate per 1000 person tested) for infectious syphilis by sexual orientation among women and heterosexual men in major cities of Australia attending sexual health clinics: 2011-2019 (n=52,221)

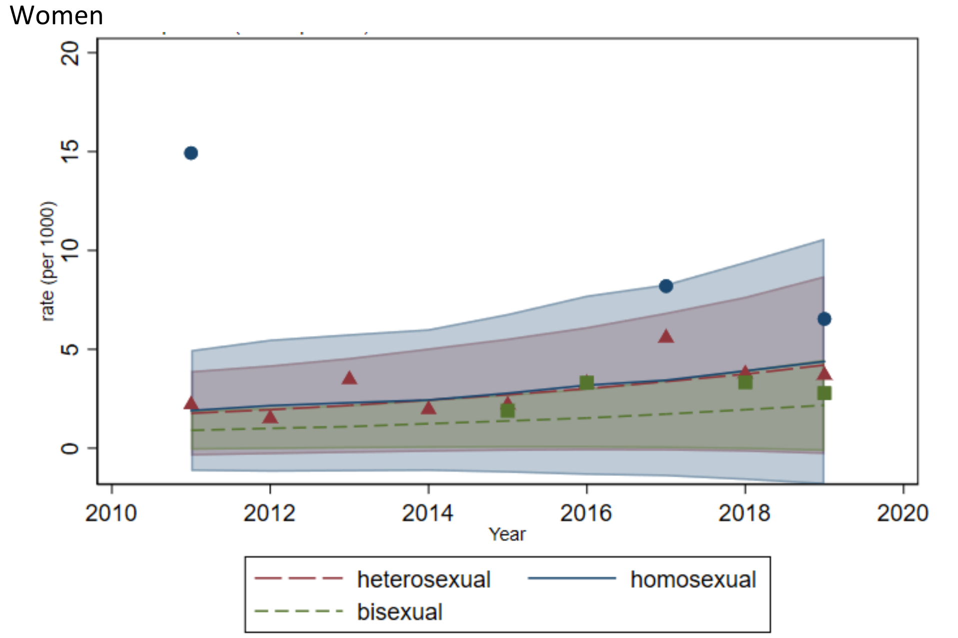

	2011	2012	2013	2014	2015	2016	2017	2018	2019
Heterosexual	2.20	1.51	3.48	1.96	2.19	3.29	5.58	3.76	3.69
Homosexual	14.9	(-)	(-)	(-)	(-)	(-)	8.20	(-)	6.54
Bisexual	(-)	(-)	(-)	(-)	1.90	3.31	(-)	3.34	2.78

Note: Scatter plot represents observed rates. Line plot shows fitted mean annual rate of change, adjusted for patient-level random effects and annual fluctuation.

Figure 7. Annual positivity (rate per 1000 person tested) for infectious syphilis by sex worker status among women and heterosexual men in major cities of Australia attending sexual health clinics: 2011-2019 (n=52,221)

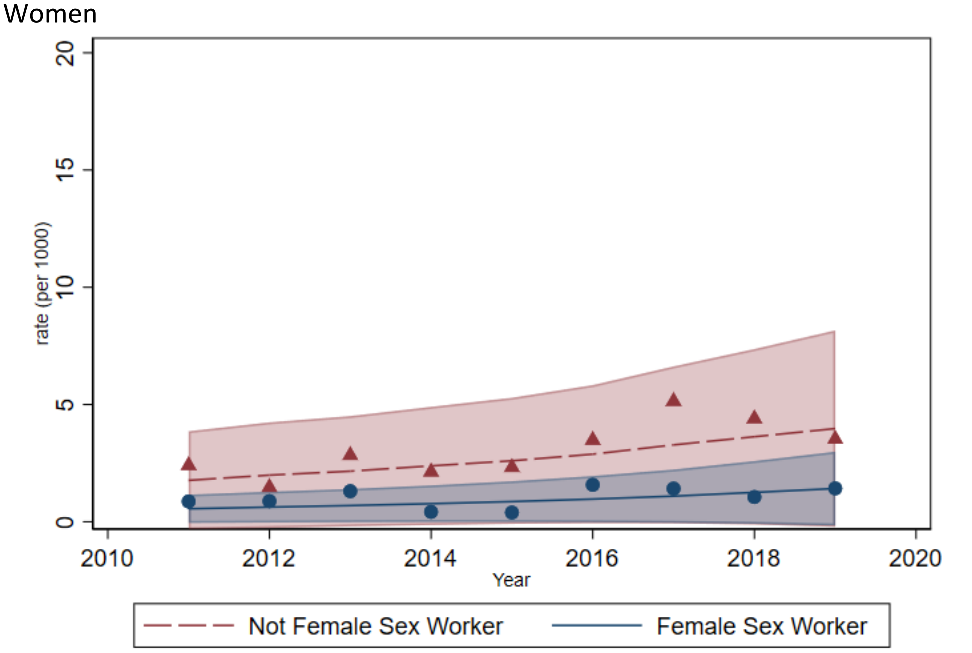

	2011	2012	2013	2014	2015	2016	2017	2018	2019
Sex worker rates	0.87	0.88	1.30	0.42	0.39	1.58	1.42	1.06	1.42
Non-Sex worker rates	2.40	1.47	2.83	2.13	2.32	3.48	5.14	4.40	3.52

Note: Scatter plot represents observed rates. Line plot shows fitted mean annual rate of change, adjusted for patient-level random effects and annual fluctuation.

Table. Annual positivity (rate per 1000 persons tested) for infectious syphilis by sex and characteristic

A. Women

	Year								
**Characteristic**	2011	2012	2013	2014	2015	2016	2017	2018	2019
Indigenous status									
Aboriginal or Torres Strait Islander	* (18.9)	0 (-)	* (15.4)	* (15.2)	* (-)	* (-)	* (10.0)	* (11.2)	* (47.1)
Non-Indigenous	10 (1.67)	8 (1.27)	15 (2.20)	11 (1.47)	13 (1.70)	22 (2.88)	31 (3.79)	33 (3.37)	28 (2.62)
Culturally or linguistically diverse background									
Yes	* (1.48)	* (1.08)	7 (2.38)	* (1.21)	7 (1.98)	9 (2.51)	* (0.99)	9 (1.87)	9 (1.70)
No	7 (2.09)	5 (1.39)	9 (2.28)	8 (1.89)	6 (1.44)	13 (3.16)	28 (6.60)	25 (4.92)	23 (4.22)
Arrival in Australia									
Born in Australia	6 (2.33)	5 (1.93)	9 (3.25)	7 (2.34)	7 (2.51)	12 (4.26)	24 (7.45)	26 (6.80)	24 (5.92)
Less than two years	* (1.26)	* (0.57)	* (1.92)	* (0.42)	* (0.38)	* (1.56)	* (1.90)	* (0.97)	* (0.92)
Two to less than five years	* (2.22)	* (-)	* (2.33)	* (-)	* (1.89)	* (1.70)	* (-)	* (1.90)	* (2.10)
Five years or more	* (1.01)	* (1.87)	* (0.84)	* (3.20)	* (2.42)	* (3.46)	* (2.60)	* (1.45)	* (0.65)
Socio-economic status (IRSAD centile)									
81–100 [least disadvantage]	* (1.61)	* (1.14)	* (0.35)	* (1.26)	7 (2.18)	6 (1.83)	13 (3.73)	16 (3.68)	12 (2.59)
41–80	* (0.41)	* (0.76)	7 (2.40)	5 (1.60)	* (0.90)	10 (3.08)	9 (2.59)	10 (2.44)	13 (2.84)
0–40 [greatest disadvantage]	6 (5.46)	* (2.72)	8 (7.10)	* (2.43)	* (2.56)	6 (5.08)	10 (7.58)	8 (5.56)	7 (4.51)
Injection drug use in the past twelve months									
No	10 (7.19)	8 (-)	14 (11.56)	12 (-)	11 (12.7)	18 (25.5)	29 (19.2)	30 (29.4)	27 (27.6)
Yes	* (1.69)	* (1.29)	* (2.08)	* (1.63)	* (1.46)	* (2.38)	* (3.57)	* (3.08)	5 (2.55)

	Year								
**Characteristic**	2011	2012	2013	2014	2015	2016	2017	2018	2019
Sexual orientation									
Heterosexual	8<br>(2.20)	6<br>(1.51)	15<br>(3.48)	9<br>(1.96)	10<br>(2.19)	15<br>(3.29)	28<br>(5.58)	23<br>(3.76)	24<br>(3.69)
Homosexual	*<br>(14.9)	*<br>(-)	*<br>(-)	*<br>(-)	*<br>(-)	*<br>(-)	*<br>(8.20)	*<br>(-)	*<br>(6.54)
Bisexual	*<br>(-)	*<br>(-)	*<br>(-)	*<br>(-)	*<br>(1.90)	*<br>(3.31)	*<br>(-)	*<br>(3.34)	*<br>(2.78)
Sex work in the past twelve months									
Yes	*<br>(0.87)	*<br>(0.88)	*<br>(1.30)	*<br>(0.42)	*<br>(0.39)	*<br>(1.58)	*<br>(1.42)	*<br>(1.06)	*<br>(1.42)
No	9<br>(2.40)	6<br>(1.47)	13<br>(2.83)	11<br>(2.13)	12<br>(2.32)	18<br>(3.48)	28<br>(5.14)	31<br>(4.40)	28<br>(3.52)

* Values < 5.

B. Men

	Year								
**Characteristic**	2011	2012	2013	2014	2015	2016	2017	2018	2019
Indigenous status									
Aboriginal or Torres Strait Islander	* (-)	* (-)	* (-)	* (-)	* (17.2)	* (-)	* (-)	* (-)	* (13.3)
Non-Indigenous	22 (6.09)	9 (2.14)	22 (4.95)	19 (3.95)	25 (5.55)	25 (5.15)	29(5.79)	37 (5.81)	49 (7.50)
Culturally or linguistically diverse background									
Yes	9 (10.1)	* (1.74)	6 (4.84)	7 (5.03)	6 (4.58)	5 (3.32)	7 (4.33)	8 (3.89)	9 (4.09)
No	13 (4.75)	7 (2.27)	16 (4.94)	12 (3.45)	20 (6.15)	20 (5.86)	22 (6.33)	29 (6.64)	41 (9.29)
Arrival in Australia									
Born in Australia	12 (5.40)	7 (2.99)	16 (6.45)	12 (4.36)	16 (6.35)	14 (5.11)	20 (7.00)	28 (7.91)	40 (11.2)
Less than two years	* (5.53)	* (-)	* (1.31)	* (3.61)	* (2.60)	* (5.37)	* (2.55)	* (2.84)	* (1.76)
Two to less than five years	* (6.78)	* (-)	* (-)	* (-)	* (-)	* (4.21)	* (-)	* (1.55)	* (2.93)
Five years or more	5 (8.76)	* (2.68)	5 (5.92)	* (4.43)	8 (9.10)	5 (5.20)	7 (7.21)	5 (4.22)	6 (4.95)
Socio-economic status (IRSAD centile)									
81–100 [least disadvantage]	6 (4.40)	* (1.20)	* (2.25)	7 (3.61)	9 (4.81)	12 (6.03)	8 (3.84)	8 (3.05)	14 (4.99)
41–80	8 (5.36)	* (2.37)	8 (4.42)	6 (3.2)	12 (6.85)	7 (3.69)	14 (7.18)	17 (6.74)	21 (8.09)
0–40 [greatest disadvantage]	8 (10.3)	* (3.45)	10 (11.2)	6 (5.96)	5 (5.31)	6 (5.8)	7 (6.63)	12 (9.36)	15 (12.4)
Injection drug use in the past twelve months									
No	22 (6.18)	9 (2.17)	21 (4.87)	18 (3.81)	26 (5.93)	23 (4.84)	26 (5.27)	33 (5.27)	47 (7.28)
Yes	* (-)	* (-)	* (5.95)	* (6.80)	* (-)	* (12.1)	* (19.0)	* (25.2)	* (19)

* Values < 5.
